# Everybody's Page

**Published:** 1889-11-09

**Authors:** 


### Everybody's Page.

Cotton-wool is a gentle and safe method of applying moist warmth, and more continuous than poultices.

M. Rouxel has recently enunciated a theory that the Gauls invented the zodiac, and that it was in existence before the Flood.

There has recently been a severe epidemic of cholera in Mesopotamia and Persia, which has, however, decreased since the cold weather set in.

The reason the evening primrose is yellow is that, being a night flowering plant, fragrant in the evening, its pale yellow colour makes it easily recognisable by moths, who feed upon its honey. Spots and lines on the petals of flowers act as honey-guides.

It is not strange that the superstitious notion that the royal touch could cure scrofula existed so long, when a man in the position of Sir Thomas Wiseman, physician to Charles II., would venture to say, "I must needs admit that his Majesty cureth more in one year than all the chirurgeons of London have done in any age."

"Change of occupation is better than rest"; in fact, rest is simply a change of occupation. Rest for the body is attained by change of posture, lying in bed, instead of sitting or standing. Rest for the mind is attained in the same way, by fixing it on something different from that which has wearied it.

It is rather a pity that medicinal herbs are so much less cultivated in gardens than they used to be. Balm, peppermint, marigold, and feverfew are simple remedies, which, even when given by amateur doctors, are more likely to do good than the medicines "promiscuously applied" out of the terrible chest which so many people keep and torture their families with.

The *Sudor Anglicus*, or "sweating sickness," one of th most rapidly fatal of all the plagues of bygone days, first made its appearance in 1485. It broke out among the foreign soldiers who came over with the Earl of Richmond, and thence spread to London. It returned four times—in 1506, 1517, 1528, and 1551. The chief symptom was, as the name implies, profuse perspiration, and a fatal termination was reached within twenty-four hours, sometimes, indeed, in one or two.

Many people have thought that the frequency with which dyspepsia and other gastric complaints are met with in America is due to the large consumption of iced water, ice cream, in fact, ice in any form which is there much in favour. It is doubtful, however, if the mere temperature of these beverages is responsible for all the ills they cause. But as recent studies in bacteriology show that some microbes will not die on being exposed to the cold necessary to produce congelation, it is possible that some impurity in the water or cream used in making the ice is the cause of the dyspepsia and other troubles.

The "harmless, necessary cat" should be praised in higher terms than these. Cats are regarded as officials in the United States Post Office on account of their services in destroying the rats who would nibble at the letter-bags. When any of these official pussies adds to the feline population information is sent to the superintendent of the district, who thereupon orders additional rations. Cats are included in the *personnel* of the French State printing office, and have a man whose sole duty is to look after them; and the staff of the Midland Railway includes eight cats, who protect corn-sacks from four-footed thieves.

The sulphurous alkaline waters of Aix-les-Chapelle are very beneficial to persons suffering from skin affections, or from mercurial poisoning.

Cataract is very frequently inherited, and Carns quotes a case where all the children of a man, who suffered from cataract, were born with that disease.

Mr. Jonathan Hutchinson declares that if mankind had continued to be vegetable feeders, and never known the use of wine or beer, we should have had no experience of gout.

Dr. Kovaleski, a St. Petersburg doctor, declares that every year drunkenness increases in Russia, and holds with with Dr. Norman Kerr that it is a nervous disease common to all countries and all classes of society.

The savage races of man are akin to the lower animals in the acuteness of their sense of smell. In North America the Indians can follow their enemies or their game by the scent, and in the Antilles the maroon negroes distinguish by the scent a white man's trail from a negro's.

A writer in the early part of the eighteenth century wrote as follows about the science of medicine: "It is a very difficult science, because the theory depends upon the understanding and the practice upon the imagination. It is full of dangers to the patient, because it is founded upon conjectures; and, according to Plato, the conjectures of physicians are very uncertain."

A "Weekly Rest Congress" has recently been held under the presidency of M. Leon Say, and it has expressed its conviction that one day of rest in every seven is necessary for the health of man. Mr. Gladstone's testimony was quoted: "that he attributes the prolongation of his life in great part to the care he has always taken to observe the rest of Sunday." Sunday is certainly the most convenient rest day for most people, and it is well, in order to make family gatherings easy, that all members should have their holiday on the same day. But those who cannot have Sunday rest should have 52 days at as regular intervals as possible.

The number of emigrants who landed at New York during last year was 370,822, a large majority of whom, according to the record of their destination entered in the report of the Commissioners of Emigration, meant to remain in the State of New York. These numbered 150,270; Pennsylvania came next with 46,105, while Illinois was favoured with 29,845. The two Carolinas seemed to be the least popular of the States; only 89 passengers meant to go to South Carolina and 69 to North, while 2,973 were bound for Texas, 146 for Idalio, 177 for Nevada, and 214 for the Indian territory. The greater number of the emigrants came from Germany, no fewer than 78,145. Ireland came next with 44,307, though Italy was not far behind with 43,927. In fact, more men came from Italy than from the Green Isle, the proportion being 34,556 men to 9,371 women, while among the Irish the proportion of the two sexes approximated more closely than among the emigrants of any other nationality—23,697 males to 20,610 females. Of England's 38,355 there were 24,917 males more than the Irish male contingent, but only 13,438 females. Sweden sends 37,934 emigrants to swell the great republic, and Russia 33,052. The country least given to emigration is Portugal, which last year contributed a total of three emigrants—two men and one woman. There is therefore as yet small need of a Portuguese emigrant society, such as exists for the Irish and German, with a president who is *ex officio* a member of the Board of Emigration.

